# Tactile to visual number priming in the left intraparietal cortex of sighted Braille readers

**DOI:** 10.1038/s41598-020-72431-7

**Published:** 2020-10-16

**Authors:** Katarzyna Rączy, Maria Czarnecka, Małgorzata Paplińska, Guido Hesselmann, André Knops, Marcin Szwed

**Affiliations:** 1 Department of Psychology, Jagiellonian University, Krakow, Poland; 2 The Maria Grzegorzewska University, Warsaw, Poland; 3 Department of General and Biological Psychology, Psychologische Hochschule Berlin, Berlin, Germany; 4 LaPsyDÉ, UMR CNRS 8240, Université de Paris, Paris, France

**Keywords:** Cognitive neuroscience, Sensory processing

## Abstract

Numbers can be presented in different notations and sensory modalities. It is currently debated to what extent these formats overlap onto a single representation. We asked whether such an overlap exists between symbolic numbers represented in two sensory modalities: Arabic digits and Braille numbers. A unique group of sighted Braille readers underwent extensive Braille reading training and was tested in an fMRI repetition-suppression paradigm with tactile Braille digit primes and visual Arabic digit targets. Our results reveal cross-modal priming: compared to repetition of two different quantities (e.g., Braille “5” and Arabic “2”), repetition of the same quantity presented in two modalities (e.g., Braille “5” and Arabic “5”) led to a reduction of activation in several sub-regions of the Intraparietal Sulcus (IPS), a key cortical region for magnitude processing. Thus, in sighted Braille readers, the representations of numbers read by sight and by touch overlap to a degree sufficient to cause repetition suppression. This effect was modulated by the numerical prime-probe distance. Altogether this indicates that the left parietal cortex hosts neural assemblies that are sensitive to numerical information from different notations (number words or Arabic digits) and modalities (tactile and visual).

## Introduction

The cognitive and neuronal mechanisms through which numbers are perceived, represented, and processed are controversial. It is currently debated whether the analogue magnitude code in a key cortical region for numerical magnitude processing—the Intraparietal Sulcus (IPS)—is truly abstract. This abstract type of representation has been proposed in the Triple Code Model^1^. Several studies to date have demonstrated that numbers are indeed represented in the IPS in an abstract way/manner, regardless of whether they are presented symbolically or non-symbolically (notation), simultaneously or sequentially (presentation format) or in a visual or auditory modalities (sensory modality^2–8^). Thus, activation within the IPS is greater for numbers than letters or colors^4,7^, as subjects compare the numerical or physical size of Arabic number symbols or their brightness^9,10^ then other non-numeric stimuli. Also, numerical quantity information, regardless of notation is processed in the same neural circuits within the IPS^6,11^.

While these studies imply that IPS contains a supramodal number representation^4,7^, others cast doubt on the assumption of a purely abstract magnitude representation within this cortical region^12^. Notably, two studies recently showed coding of numbers depending on the format, i.e. numerals presented as dot patterns (non-symbolically) and Arabic numerals (symbolically)^13,14^. Regardless whether a classifier was trained on the symbolic or non-symbolic format the characteristic voxel patterns that allowed the classification of numerals within a given format were format-specific and did not allow decoding across formats. In another fMRI experiment^12^, participants were presented with a series of beeps (auditory) and a series of dots (visual). Neither the auditory nor visually presented sequences elicited number-related parietal activity; however, such activation was observed in sensory areas as supported by successful decoding. Importantly, by carefully separating numerical processing from response-related processes, this study highlights the potential confound between active response preparation and selection processes and number processing in previous studies. These findings bring into question the notion of a supramodal number representation. A central debate in the number processing research is therefore whether the neural numerical magnitude representation is truly abstract i.e., modality-independent, or whether the IPS contains partially distinct representations for different modalities (e.g., tactile vs. visual) that do not indicate a purely abstract representation of numbers.

According to the Triple Code Model, numerical information is represented by three codes that are separate but interacting with each other. A verbal number code, located within the left perisylvian language areas and left angular gyrus (AG), becomes activated in tasks such as number naming, counting, or retrieving arithmetic facts from long term memory i.e. in operations where language mediation is necessary. A visual number code in the bilateral fusiform and lingual regions of the ventral visual stream allows for number words, Arabic digits and multi-digit numbers recognition. Finally, an analogue magnitude code in the IPS is assumed to represent numerical quantity information^15,16^ in an approximate manner. If this notion is correct, i.e. the numerical representation within IPS is indeed truly abstract, then the same neural population would be engaged in encoding of numerical quantity, regardless of the modality (tactile or visual). The activation of the latter would indicate that a truly abstract magnitude representation would be potentially accessible via multiple modalities, that is, via visual, auditory, or tactile input.

We reasoned that a repetition study of cross-modal number priming in a unique population of sighted adults who read both by touch and by sight^17–20^ would be particularly informative. Repetition suppression in number processing refers to a decreased neural response to a stimulus when the prime stimulus that is similar (e.g., three) precedes target stimulus (e.g., “3”). Repetition suppression reflects a facilitation of the targets’ processing, due to the overlapping representation between the prime and the target. We reasoned that repetition of the same quantity presented in two modalities (e.g., tactile Braille “5” and visual Arabic “5”) should lead to a stronger reduction of activation in the IPS, compared to two different quantities (e.g., Braille “5” and Arabic “2”). If the representation for numbers is fully abstract, adaptation across two different modalities (tactile to visual modality) should be observed within the IPS of sighted Braille readers.

## Materials and methods

### Participants

Twenty-four females (mean age = 26, range 23–32) participated in the experiment. One participant was excluded due to the extensive head movements during fMRI acquisition. All participants were right handed, monolingual with normal or corrected to normal vision. The participants were students or college graduates with a minimum of 3 years of higher education (mean = 5 range 3–12 years of education). Similar to our previous study^21^ the group was recruited from participants of two previous projects, performed in 2016 and 2017, which included a 9-months Braille reading course. During that Braille course, participants acquired tactile recognition of all the Braille letters in the Polish alphabet (for details see^18^). For the purpose of the current experiment all participants attended a 3-week course focused specifically on reading Braille numbers. The course took place in May 2018, i.e., either one or two years after the main, 9-months course. The course was designed and carried out by an experienced Braille teacher who also had co-designed the previous course (M.P., a co-author of this article). Since numbers in the Braille alphabet are letter symbols from A to J preceded by a number indicator, participants needed only three-weeks to automatically recognize Braille numbers. Similar to the word reading course, the number course relied primarily on participants’ individual work. At its onset participants received 20 exercises, each printed on a single sheet. They were asked to complete one exercise per day while blindfolded, and then to check their results visually. During the first two weeks of the course, twice a week, a class was organized during which participants were given a subsequent set of Braille exercises, received detailed and personalized instruction about completing them, practiced a sample set of exercises in the class and received personalized feedback from the instructor. During the second week, they met individually with the Braille teacher to resolve any potential issues (e.g., regarding proper Braille reading technique) and practice on the Braille display used in the main experiment. Despite being right-handed seven out of 23 subjects read with their left hand. This preference towards reading Braille with the non-dominant hand, is a common and well-described phenomenon in blind people^22^ and has been also reported in sighted Braille readers^18^. The study was approved by the Jagiellonian University Ethics Committee. A written informed consent was obtained from all subjects before the experiment. The methods were carried out in accordance with the relevant guidelines and regulations. Participants were reimbursed for taking part in the study.

### Behavioral tests

We tested participants’ reading speed both in visual and tactile Braille, and in the Latin alphabet. Both were tested before and after the Braille course. Four different tests were used to assess the participants’ Braille reading speed. Since there are no standardized tests measuring tactile or visual Braille reading speed in Polish we created a tactile Braille reading test of single numbers (consisting of 6 rows of Braille numbers), a tactile Braille reading test of single letters (consisting of 6 rows of single Braille letters in the Polish alphabet), a tactile Braille reading test of whole words (consisting of 4 rows of 4–6 letter long words, printed in tactile Braille), and a visual Braille reading speed test (consisting of 30 words, 4–6 letter long, printed in visual Braille font). To test participants’ visual reading speed we used a test designed by Bola et al.^18^, which consists of a 400-word passage from the book “Farsa Panny Heni” by Maria Rodziewiczówna. We tested the visual reading speed only once, before the Braille course. Additionally, before and after Braille course, we tested participants’ tactile acuity of the index finger in the reading hand with the tactile acuity grating orientation task^23^. Since differences between reading Braille on paper vs. on a digital display exist, for the purpose of the main experiment we tested the participants’ ability to recognize Braille numbers on a Braille display (https://www.harpo.com.pl/) by asking them to read aloud a Braille number (range 0–9; 20 repetitions per number) that appeared on the Braille display for a varying amount of time (300 ms, 600 ms or 900 ms).

### Stimuli and procedure

**Figure 1:**
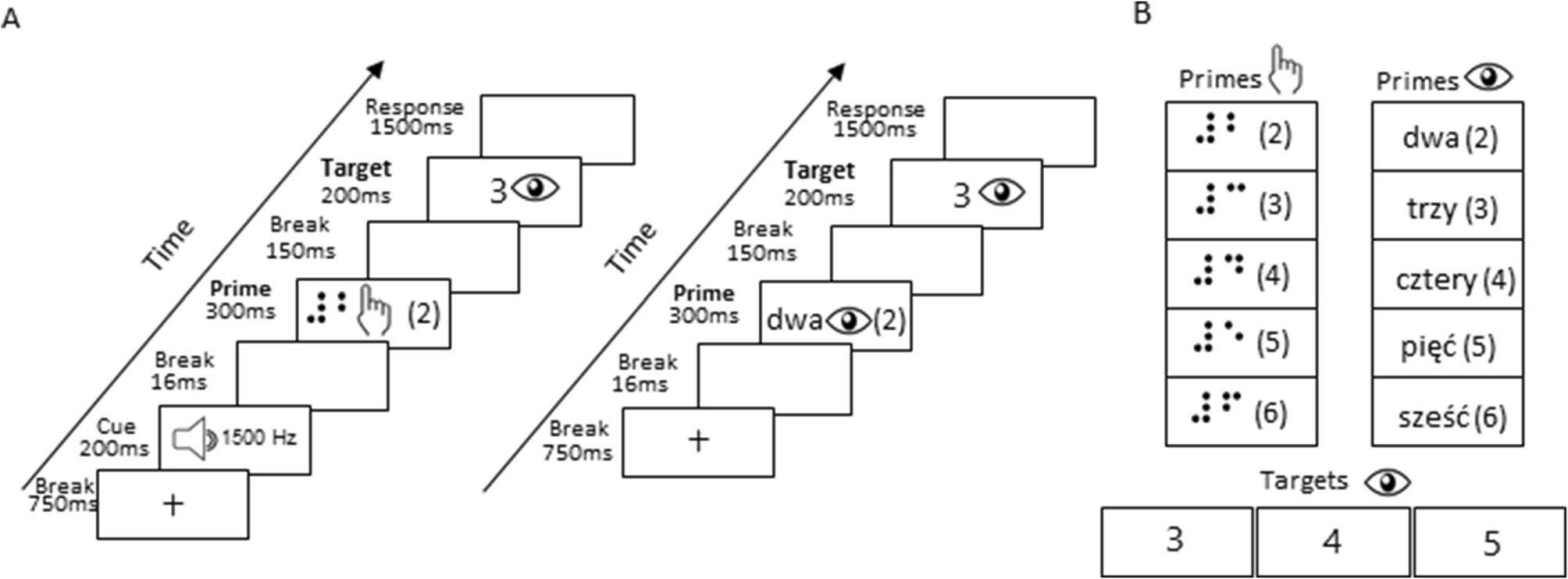
Experimental paradigm. (**A**) Shows tactile Braille digits to visual Arabic digits priming and number words to Arabic digits priming. (**B**) Shows stimuli: tactile primes, visual primes and visual targets used in the experiment. Participants were asked to indicate the quantity of the target Arabic number displayed on the computer screen and press the corresponding button.

Figure 1B shows the stimuli used in the experiment. The stimuli were based on Roggeman et al.^24^, Reynvoet and Brysbaert^25^, and Reynvoet et al.^26^ and were identical to those used in our recent behavioral experiment^21^. Following our previous experiment^21^, we excluded the number 1 from the stimuli to avoid the obvious correspondence—one dot = number 1. We used only one-digit numbers from the range 2–6 for the three main reasons: (1) In Braille alphabet numbers are letters preceded by a number indicator. The number indicator does not have to be actively read by the participant i.e. it is sufficient to “feel” the dots on the edge of the fingertip of the reading finger. In result one-digit numbers do not have to be actively swept through to be read, contrary to two-digit numbers that require moving the reading finger through at least two slots on the Braille display. The recognition of a two-digit number in the current experiment might have therefore significantly increased reading time. As consequence, it might have increased SOA and influenced the priming effect. (2) There are substantial differences between visual and tactile reading: contrary to mature sighted reading—i.e. one word at a time (no within-word saccades),—tactile reading is sequential. To control for listed differences between visual and tactile reading systems, we decided to use only one-digit numbers in the current experiment. 3) In Braille alphabet numbers 2, 3, 5 are two-dot numbers, 4, 6, 8, 0 are tree-dot numbers and 7 is a four-dot number. Thus, in order to have a continuous set of number-stimuli (and at least five different primes), with the same number of dots, we decided to use number stimuli from 2 to 6 in the current experiment. We tested two conditions: (1) tactile Braille digits to Arabic digits number priming and (2) number words to Arabic digits number priming. Consequently, primes were either tactile Braille numbers (i.e., a letter preceded by a number indicator, a combination which stands for a number in the Braille alphabet) displayed on the Braille display (a custom made fMRI-compatible Braille display https://www.neurodevice.pl/) or number words presented visually. Targets were always Arabic digits (range from 3 to 5) presented in white on a black background. Number words and Arabic digits were presented in Courier font with a font size comparable to the Braille numbers (font size: 36) foveally on a 32-inch LCD monitor (60-Hz refresh rate) and viewed by the subjects via a mirror.

Participants were asked to indicate the numerosity of the Arabic digit displayed on the screen by pressing the corresponding button. A Wireless S2 Ergo Pad with four buttons was used (https://smit-lab.eu/). The response pad has a grip with four buttons: under the thumb, the index finger, the middle finger and the ring finger. In our experiment each button corresponded to the stimuli used in the experiment: 2, 3, 4, 5, respectively. In the case of the number 6 (in the catch trials) participants were asked not to press any button. Since the response pads used in the experiment are wireless, participants practiced number-button correspondence before the fMRI experiment. Additionally, to test whether the participants paid attention to the tactile primes and to test their recognition, in each run, subjects were presented with additional “catch trials”. In these trials, after a regular tactile trial a question mark followed by a response pad picture appeared and participants were asked to indicate the quantity of the Braille number prime that preceded the target by pressing the corresponding button and in the case of “6” not to press any button.

### fMRI Experiment: experimental design

We used an event-related design. The experiment had five runs. In each run, eighty trials were presented at a rate of one trial every 5116 ms. To introduce the jitter necessary in event-related paradigms, in each run we also presented 16 additional “blank trials”, during which nothing was displayed either on the Braille stimulator or the screen for the entire 5116 ms (identical to the length of the trial), and similar to other studies (e.g.^11,27–29^).

In addition, in each run we presented the participants with catch trials (see “Stimuli and procedure”) at a rate on one catch trial every 7316 ms.

Each trial began with 750 ms break, then only in the tactile-to-visual trials, it was followed by an auditory cue (200 ms beep) indicating a tactile prime (1,500 Hz) followed by a 16 ms blank. Then, a prime was presented for 300 ms, followed by a 200 ms blank, a 200 ms target. Participants had 1500 ms to respond. After that time there was an additional 2000 ms blank (Fig. 1A). In catch trials, the blank screen was followed by the presentation of a question mark for 200 ms followed by a response pad picture which required participants to indicate the numerosity of the prime that preceded the target within a 2000 ms time frame. In each run, trials were presented in a pseudorandom order.

### fMRI acquisition

All fMRI data were acquired at the Laboratory of Brain Imaging (LOBI) in Warsaw on a 3 T Siemens MAGNETOM Trio scanner. Functional MR scans were collected using an EPI sequence. A 32-channel head coil was used (flip angle = 90°; TR = 1500 ms; TE = 53 ms, FOV = 192 mm, 32 × 32 matrix). Twenty-eight interleaved axial slices (thickness 3.5 mm; in-plane resolution = 3.0 × 3.0 mm^2^) were acquired. 3D T1-weighted MPRAGE images (resolution 1 × 1 × 1 mm^3^) were also acquired for each subject.

### fMRI data analysis

All fMRI data were analyzed using the SPM12 software package (https://www.fil.ion.ucl.ac.uk/spm/software/spm12/). A standard pre-processing pipeline was used in which all the acquired functional volumes were corrected to the first slice for EPI distortion and slice acquisition time; they were subsequently realigned using rigid body transformations to correct for head movements, normalized to the standard adult template (MNI space), and smoothed with a 6 mm (FWHM) Gaussian kernel. The hemodynamic activity for each condition (*Same, Different)* and each modality *(tactile, visual)* and six estimated movement parameters as regressors were first modeled within a general linear model for each subject. In the second level analysis, we carried out a random-effects ANOVA.

Since the repetition suppression is constrained to the unique population of neurons that responds to certain stimuli, and its effects are relatively weak (see^30^ for a general discussion) in all contrasts, we applied a voxel-wise threshold of p < 0.001, corrected for multiple comparisons across the whole brain using a FWE-corrected cluster extent threshold of p < 0.01, similar to other repetition studies (e.g.^27–29,31,32^).

## Results

### Behavioral tests

In all reading speed tests we report the number of correctly read numbers/letters/words in the allotted time. At the onset of the three-week Braille course, the mean tactile single Braille number reading speed among the participants was 10.83 numbers per minute (NPM) (SEM = 1.33 range 3–28). The mean tactile single Braille letter reading speed among the participants was 15.22 letters per minute (LPM) (SEM = 0.98 range 7–26). The mean tactile Braille word reading speed among the participants was 4.88 words per minute (WPM) (SEM = 0.63 range 1–14). The mean visual Braille word reading speed among the participants was 19 words per minute (WPM) (SEM = 1.38 range 7.5–35).

After the Braille course participants reached an averaged performance of 28.83 numbers per minute (NPM) (SEM = 2.46 range 6–49), 18.43 letters per minute (LPM) (SEM = 1.17 range 9–32), 6.10 words per minute (WPM) (SEM = 0.71 range 1–16) in the tactile modality and 21.63 words per minute (WPM) (SEM = 1.36 range 9.5–37.5) in the visual modality.

To directly test for an increase in the Braille reading speed, we ran paired t-tests between ‘before’ vs. ‘after’ course data from all the tests described above. These analyses confirmed that the increase in Braille reading speed was significant in all tests (tactile Braille numbers: t(19) = − 8.586, p < 0.001; tactile Braille letters: t(19) = − 4.948, p < 0.001; tactile Braille words: t(19) = 3.751, p < 0.001; visual Braille words: t(19) = − 5.197, p < 0.001). Thus, the results show a significant increase in the visual Braille reading speed as well.

The subjects’ reading speed of visual text with full sentences (“Farsa Pani Heni”) was 162.25 words per minute (WPM) (SEM = 7.79; range 91–229) which is a standard result for visual reading speed in skilled adults (e.g.^18,33^). The subjects’ visual reading speed was not correlated with any of the tests measuring Braille reading speed (tactile Braille numbers: r(20) = − 0.169, p = 0.442; tactile Braille letters: r(20) = − 0.152, p = 0.487; tactile Braille words: r(20) = − 0.102, p = 0.643; visual Braille words: r(20) = − 0.309, p = 0.151).

We also measured the subjects’ tactile acuity with the tactile acuity grating orientation task^23^. At the onset of the Braille course, the mean grating orientation threshold for the reading finger was 2.03 mm (SEM = 0.16) and at the end of the course it reached the level of 1.72 mm [SEM = 0.16; which represents a significant improvement t(22) = 4.575, p < 0.001].

Since differences between reading Braille on paper vs. on a digital display exist, additionally for the purpose of the main experiment we tested participants’ ability to recognize Braille numbers on the digital Braille Display. On average participants recognized 85% (SEM = 2.34, range 14–20) Braille numbers when displayed for 900 ms, 73% (SEM = 4.31, range 4–19) Braille numbers when displayed for 600 ms, 71% (SEM = 3.44, range 10–20) Braille numbers when displayed for 300 ms. Thus, in the main experiment we displayed the prime for 300 ms.

### Behavior in the scanner

**Figure 2:**
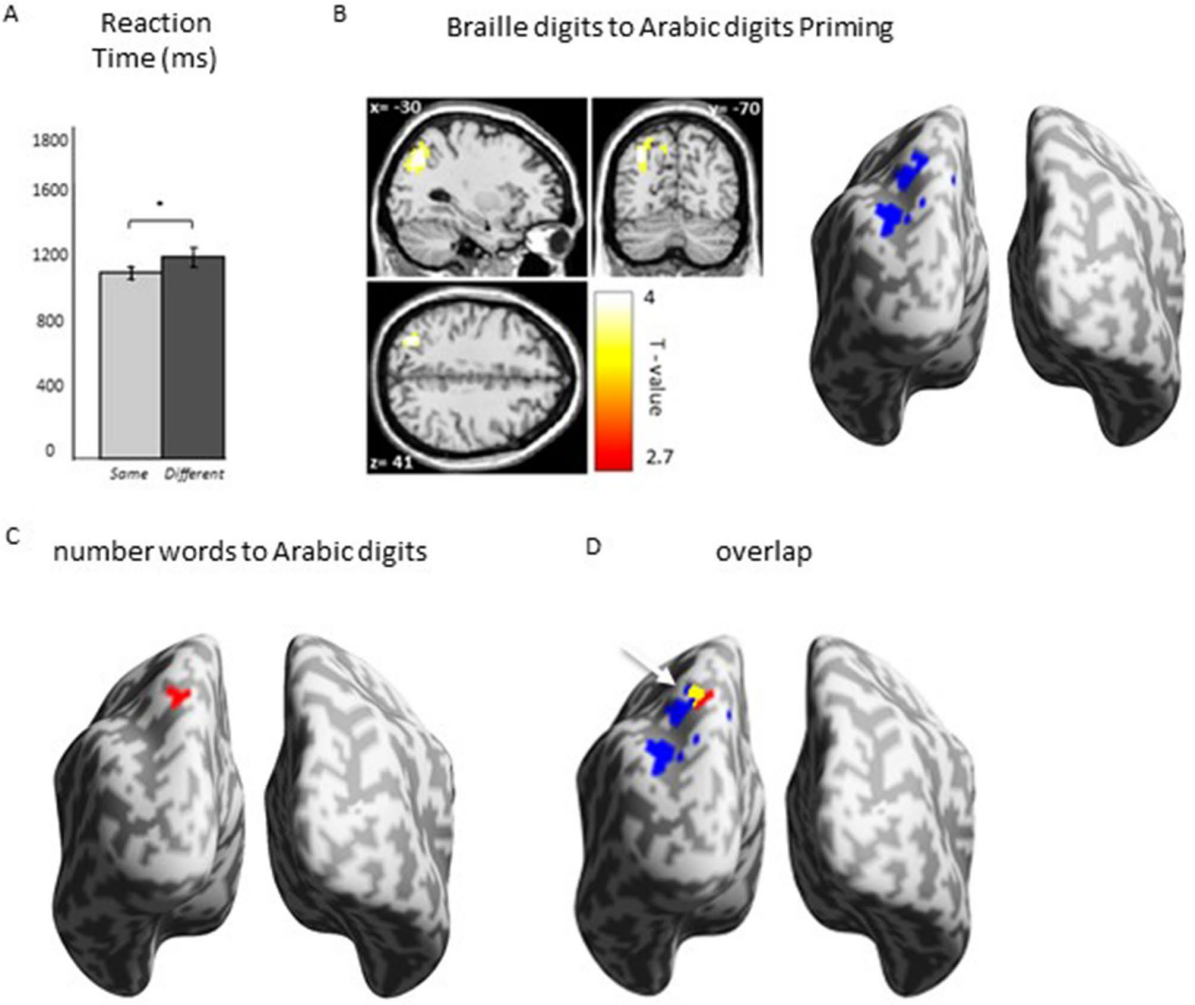
Results. (**A**) Behavioral Results in the scanner. Reaction Times for Different > Same condition. Significance level: *p < 0.01. Error bars represent S.E.M. (**B**) Whole-brain analysis. Repetition suppression for numerical repetition priming with tactile primes (Braille digits) to visual targets (Arabic digits) in Different vs. Same condition. Threshold p < 0.001 voxel-wise (uncorrected), p < 0.01 cluster-wise (FWE-corrected). (**C**) Number words to Arabic digits number priming showed a rather weak effect within the left IPS, however occurred in a location previously reported for visual number priming (e.g.^11^). (**D**) overlap indicating at least partial overlap of the tactile and visual number code.

The participants’ accuracy in the task (in which participants indicated the numerosity of the Arabic digits displayed on the screen by pressing a corresponding button) was 96.42% (SEM = 4.69) in the tactile number priming and 95.07% (SEM = 1.39) in the visual number priming. In the catch trials (where the participants had to additionally indicate the tactile prime that preceded the target) the participants’ accuracy was 82.33% (SEM = 9.64). We also measured Reaction Times for *same* and *different* conditions for both tactile-to-visual and visual-to-visual number priming to establish whether there was a priming effect observed on the behavioral level (e.g.^31,32,34,35^). A paired t-test between *same* vs. *different* conditions, revealed a significant difference for tactile-to-visual number priming (1081 and 1117 ms for *same* and *different* condition respectively; t(22) = -3.01, p = 0.007), see Fig. 2A) and for visual-to-visual number priming (971 and 1053 ms for *same* and *different* condition respectively; t(22) = -5.47, p < 0.001).

### Whole-brain fMRI analysis

If number representation within the IPS is shared for different sensory modalities, our prediction was that repetition of the same quantity across two different modalities (i.e., tactile and visual) will cause reduced activation within the IPS relative to non-repeated quantities. To test for these repetition suppression effects, we conducted a whole-brain analysis. We compared activations induced by *Different* > *Same* conditions for the tactile-to-visual number priming. This contrast revealed a robust activation in the left IPS (Peak MNI = -30 -70 41, t = 4.44, cluster-level extent threshold p < 0.01, FWE-corrected, cluster size k = 118 voxels, Fig. 2B). This cluster had two sub-peaks: MNI = -9 -73 45 and MNI = -18 -73 55. Then we compared activations induced by *Different* > *Same* conditions for the visual-to-visual number priming. This contrast revealed a small activation in the left IPS (Peak MNI = -18 -70 55, t = 4.44) but below cluster-level extent threshold (cluster-level extent threshold p = 0.49, FWE-corrected, cluster size k = 20 voxels, Fig. 2C).

Additionally, to further test whether the observed activation reflects number-specific activation, we first compared activations induced by all trials available to us (tactile-to-visual and visual-to-visual number processing) i.e. All number trials > Rest contrast in our experiment (see Supplementary Fig. 1A). The results resembled typical activations for number processing tasks (e.g.^4,6,13,14^). Subsequently, we created a mask of All number trials > Rest contrast, and then, analyzed the Different > Same contrast for tactile-to-visual number priming, within this inclusive mask that we created. The obtained result (see Supplementary Fig. 1B) has not changed compared to the result presented in Fig. 2B. This indicates that the IPS identified in the current study broadly supports number processing.

Our study shows significant priming for tactile Braille to Arabic digits number priming (Fig. 2B) and a small effect of visual number words to Arabic digits number priming (Fig. 2C). The dorsal part of the cluster in the tactile-to-visual number priming (with the sub-peak MNI = -18 -73 55) overlaps with the activation shown in visual-to-visual number priming (depicted in yellow in Fig. 2D) and activation demonstrated in other cross-notation repetition suppression studies within the visual modality e.g., Naccache and Dehaene^11^ (MNI = − 44 − 56 56), Notebaert et al.^36^ (MNI = − 38 − 49 46). This overlap suggests at least partial overlap of the tactile and visual codes.

### Effect of target-prime distance

**Figure 3:**
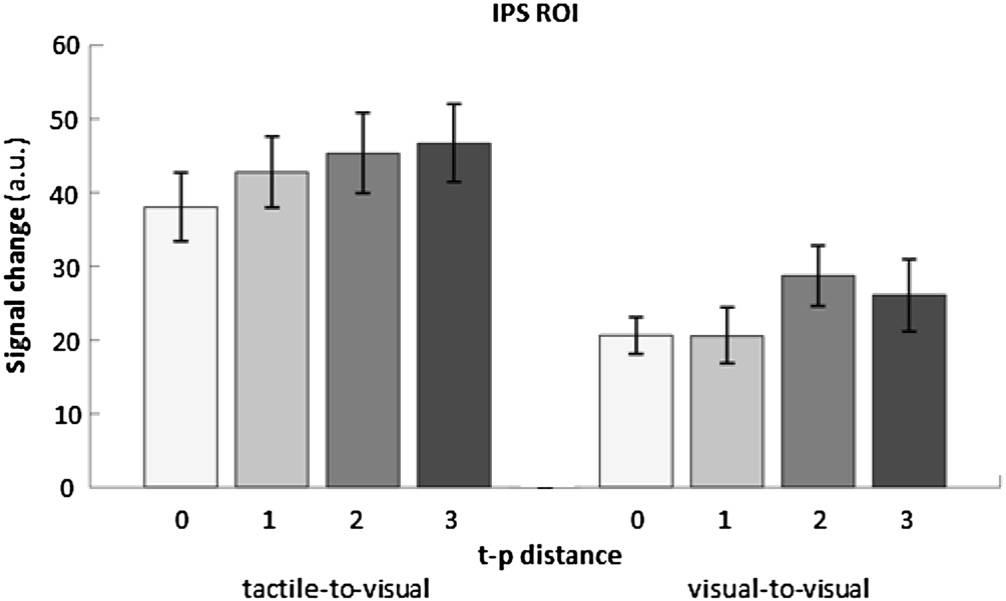
ROI analysis. Signal change (in arbitrary units, a.u.) in the IPS ROI in four target-prime (t-p) distances (0, 1, 2, 3) in tactile-to-visual and visual-to-visual number priming is shown. A 2 × 4 repeated-measures ANOVA on the individual signal change within the IPS ROI with the factors prime modality and t-p distance revealed a significant effect for modality F(1,22) = 21.42, p < 0.001, a significant effect for t-p distance F(2,66) = 4.31, p = 0.008 and a non-significant interaction F(3,66) = 0.41, p = 0.750. Errorbars represent MSE.

To further understand what drives the observed categorical priming effect, we regrouped trials according to the absolute numerical distance between target and prime (t-p distance). With our design we obtained four t-p distances (0, 1, 2, and 3) that we used to model our data. If the prime activates a position on the mental number line^11,24^, target processing should be facilitated, leading to the reduced activation upon target presentation that we observed in the *different* > *same* contrast. The extent of this reduction should be maximal for t-p distance 0, and should decrease as the absolute distance between target and prime increases. To test for the distance related effects we first defined an ROI in the IPS. To this aim, and to avoid “double dipping”^37^, we used data from another experiment on number processing (Czarnecka et al., in prep, for the full description please see Supplemental Materials) where the same subjects were presented with numerosities 2, 4, 6 and 8 in three formats: visual symbolic, visual non-symbolic and tactile symbolic (12 conditions). Stimuli were displayed on the MRI-compatible computer screen and in case of Braille numbers displayed on the Braille display. To define the IPS ROI, we contrasted all number formats (all 12 conditions) versus rest. In each subject, we defined the 100 voxels that were the most active within anatomically defined boundaries of the IPS region (non-contiguous voxels constrained by a box with anatomical boundaries: zmax = 60; zmin = 30; ymin = − 80; ymax = − 40; xmin = − 50; xmax = 40) that was large enough to encompass the activations previously published in the literature^2,4,6,7^ and constrained by the anatomical mask (using xjView toolbox https://www.alivelearn.net/xjview). We then extracted the parameter estimates (beta) from these voxels for each subject in all experimental conditions of interest (four t-p distances—regrouped trials). Separately for each subject, the activations were averaged across all 100 voxels for each condition and entered into a repeated measures ANOVA. The activation values (regression coefficient estimates) reported in ROI plot (Fig. 3) are shown in arbitrary units (beta) proportional to BOLD activation percentage.

We found a linear decrease of prime-related reduction as a function of t-p distance for Braille to Arabic digits priming (Fig. 3).

A 2 × 4 repeated-measures ANOVA on the individual signal change within the IPS ROI with the factors prime modality and t-p distance revealed a significant effect for modality F(1,22) = 21.42, p < 0.001, a significant effect for t-p distance F(2,66) = 4.31, p = 0.008 and a non-significant interaction F(3,66) = 0.41, p = 0.750. For tactile primes, pairwise comparisons were non-significant (all p > 0.165, FDR-corrected for multiple comparisons, Fig. 3). For visual primes pairwise comparisons were non-significant as well (all p > 0.081, FDR-corrected for multiple comparisons, Fig. 3).

Subsequently, we followed with a regression analysis to establish the difference in regression coefficients for t-p distance predictor in both prime modalities (Braille and number words). We fitted the regression equations with an intercept and a predictor which coded for a t-p distance (0, 1, 2, and 3) and then we ran the analysis for each participant separately^24,38–40^ using the equation:

$$y = b0 + b1\left( {x1} \right)$$

where: y—is the signal change in the IPS ROI, b0—is the intercept; b1—is the unstandardized B coefficient; × 1—is t-p distance predictor.

Then, we tested whether the obtained regression beta weight is positive and significantly different from zero.

**Figure 4:**
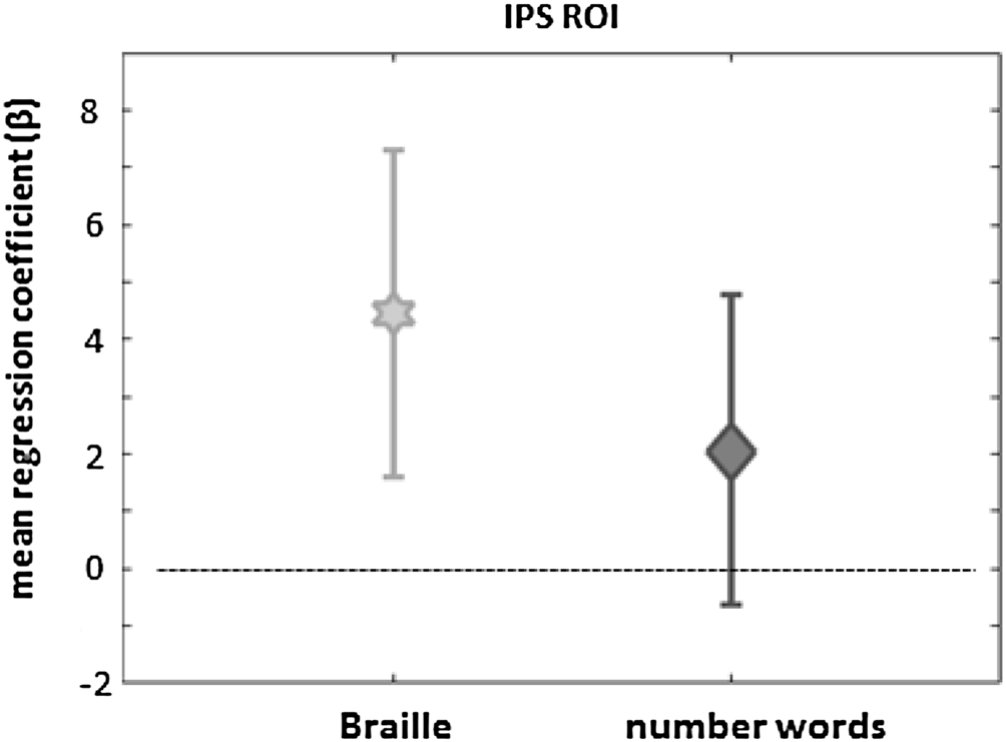
Mean regression coefficients for the target-prime (t-p) distance predictor in both prime modalities (Braille and number words). The regression was run separately for each participant for signal change arbitrary values for all 100 voxels within the IPS ROI. Errorbars denote CIs (confidence intervals).

A comparison of the t-p distance predictor with 0 revealed a significant difference for Braille primes: mean β = 2.87, MSE = 1.30, t(22) = 2.20, p = 0.039 (Fig. 4) and non-significant difference for number word primes: mean β = 2.43, MSE = 1.38, t(22) = 1.76 p = 0.092 (Fig. 4).

Together, this suggests some degree of semantic priming induced by Braille numbers that is congruent with the idea of a place coding scheme. Exploratory analyses of notation-specific effects suggest that the semantic priming is stronger for Braille primes compared to number word primes.

## Discussion

In the present study, we used a repetition priming approach to investigate whether numerical information from different modalities converges upon a common abstract number representation in the IPS. If such a representational overlap between the prime and the target across different sensory modalities existed, we should have observed a stronger reduction of IPS activation (i.e., repetition suppression), following the repetition of the same quantity presented in two modalities (e.g., as tactile Braille “5” and visual Arabic “5”) compared to two different quantities. Our study revealed two main findings: first, a significant priming effect on the behavioral level for both tactile-to-visual and visual-to-visual number priming. Second, an fMRI repetition suppression effect: the repetition of the same quantity presented in two modalities (e.g., Braille “5” and Arabic “5”) led to a reduction of activation in the IPS. This reduction was parametrically modulated by numerical distance between prime and target when the prime was a Braille number but not when the prime was a number word.

Number processing across modalities has been the subject of several previous experiments. Eger et al.^4^ presented participants with numbers, colors and letters across visual and auditory modalities and—regardless of the presentation modality—showed a bilateral IPS activation in response to numbers, but not in response to letters or colors. Subsequently, Piazza et al.^6^ demonstrated a right-lateralized IPS activation while participants estimated the comparative quantity of examples from two categories, regardless of whether these sequences were presented visually or in the auditory modality^7^. The notion of modality-independent magnitude representation has also been supported by behavioral priming studies, notably by Kouider and Dehaene^5^, in whose study participants were presented with numbers in different notations (e.g., “six” or “6”) and across different modalities (visual and auditory). Numerical repetition of the same quantity across number words and Arabic notations led to a stronger priming effect when compared to the repetition of different quantities. Such a cross-notation priming effect was supported by the fMRI repetition suppression studies of, for example, Naccache and Dehaene^11^ and Notebaert et al.^36^. Crucially, in their experiment, Kouider and Dehaene^5^ demonstrated that number priming transfers between the auditory and the visual modality, indicating that processing magnitude information requires higher-level representations that go beyond the perceptual processing stage.

At the same time, there is evidence suggesting that the way in which number-specific activation is spatially organized within the IPS does not reflect a purely abstract number representation (e.g.^12,41–44^). For example, Lyons et al.^44^ demonstrated that although the IPS is sensitive to numerosity, symbolic and non-symbolic numbers are coded within this brain region in fundamentally different ways, regardless of the presentation format. Furthermore, the study of Cavdaroglu et al.^12^ demonstrated that when the task did not require any response, neither auditory nor visual numerosities did elicit significant parietal activity. The parietal cortex, partially localized within the dorsal stream, creates a link between the visuo-spatial system and the motor system; it participates when selecting and preparing of manual and ocular actions (e.g., reaching/grasping and saccadic eye movements). Parietal activation is often observed in non-numerical tasks as participants are asked to compare two objects and increases when the comparison gets more difficult (e.g.^45^).

Our results demonstrate that repeated presentation of numerical quantity across tactile and visual modalities leads to a decreased neural response in the left IPS of sighted Braille readers. This provides an argument that numerical information across different modalities converges onto a single number representation to a degree sufficient to cause repetition suppression. This is underlined by the modulation of the effect by the numerical distance between target and prime.

Our subjects were sighted people that had learned Braille numbers while they were already fluent in recognizing Arabic digits. In a related experiment^21^ the same subjects were tested in a behavioral cross-modal priming paradigm where they performed a naming task. Participants were presented with either tactile Braille digits or number words as primes while the targets were visually presented Arabic digits, analogue to the study presented in this paper. Subjects performed a naming task. The study demonstrated priming from tactile Braille to Arabic digits that was strongest for primes of identical value (e.g., tactile Braille “4” and visual Arabic “4”). It revealed a strong effect of repetition priming, but no visible evidence for an impact of numerical distance larger than one between prime and target, thus suggesting that the magnitude information was processed according to a shared phonological code rather than a semantic code.

The impact of the tactile primes’ numerical distance on target activation in the IPS (“semantic modulation”) in the current study (Fig. 3) contrasts with these findings. Two (not mutually exclusive) discrepancies between these studies are relevant in this context. First and most obviously, the different methodologies (priming in fMRI with manual responses vs. priming outside the scanner with oral response) may render any comparison of these results difficult. Due to the different output modalities, numerical information processing stages interact with manual or phonological response codes, respectively. The parietal involvement in manual response selection may favor interactions with semantic number information in close physiological vicinity. Hence, it may be possible that sensitivity for unravelling semantic distance effects induced by the tactile primes differs between experiments. Second, the input–output compatibility (tactile—manual & visual—manual) was higher in the present experiment compared to Rączy and colleagues (tactile—vocal & visual—vocal^21^). In the context of task switching visual input paired with vocal output leads to higher switch costs compared to visual input paired with manual output^46,47^. It has been argued that this effect is due to the fact that for “compatible sensorymotor modality mappings, […] priming mechanisms facilitate response selection, resulting in reduced demands on cognitive control and reduced performance costs. In contrast, for incompatible mappings response selection is hindered because sensory input primes the compatible—but in this case not relevant—motor output. (p. 215,^48^)”. This mechanism may have facilitated the observation of semantic priming effects in the current study and/or obstrued such effects in the study by Rączy et al.^21^. Together, these factors may have favored the emergence of semantic priming effects in the current study.

Overall, priming effects were more clear-cut for tactile primes compared to visual primes (i.e. number words; see Fig. 4). Despite overall lower beta weights for the visual prime conditions, the interaction between target-prime distance and prime modality was not significant, signifying that it is not warranted to assume that the influence of the numerical distance between prime and target is modulated by prime modality. While it is difficult to draw strong conclusions from the current data set based upon a unique population of sighted Braille readers, our results conceptually replicate the findings by Dehaene and Naccache^11^ who found repetition suppression for numerical information across notations (prime: number words, target: Arabic digits) in the left parietal cortex. The question remains why we did not observe a full-blown set of statistically different priming effects at various neighboring target-prime distances for prime number words (see Fig. 3). It is well-known that lower overall activity limits sensitivity of the design which represents a limiting condition for finding more regular and robust semantic priming signature. Future studies may alleviate this issue by scanning more subjects and/or at higher SNR (e.g. due to higher field strength). Also, priming effects are notoriously dependent on stimulus timing. Since our subjects are rather slow readers (6.1 WPM, see “Behavioral tests”), which is equivalent to second grade children and slower than blind individuals who can read 60.5 WPM on average (see^27^), the timing was optimized for the tactile modality. This led to a stimulus onset asynchrony of 450 ms in the visual-to-visual condition that was considerably longer than in Roggeman et al. (132 ms) or Naccache and Dehaene (114 ms). This may have further limited the strength of semantic priming in our study, particularly affecting the number word primes. Our subjects are—to the best of our knowledge—the first systematically investigated group who is able to read in both tactile and visual domains. It remains to be proven whether equalizing their reading skills in both modalities provides even more symmetric results.

Activation in response to cross-modal priming was found in the left IPS only. This finding is supported by several other recent findings. Based on their results, Verguts and Fias^49^ propose that such asymmetry in numerical processing arises from differential specialization of the left IPS and the right IPS. While the left IPS specializes in processing number symbols, the right IPS contains representations of both notations (symbolic and non-symbolic). This notion has been supported by other reports as well: the activation in the left IPS during tasks requiring calculations on symbolic digits increases with participants’ age^50^ and right IPS activation overlaps in preschool children and adults while they process numerosity^51^. The left and right parietal lobes specialize in exact and approximate calculation, respectively^7,52–54^. While the first type of calculation either in the visual or auditory modality shows correlation with a stronger activation of bilateral parietal activation, exact judgments evoke a greater left-lateralized parietal activation compared to the right^7^. Finally, bilateral transcranial magnetic stimulation (TMS) of the IPS disrupts approximate numerical judgments, while stimulation of the left IPS disrupts the coding of precise numbers^55^. It was also demonstrated that tasks requiring comparisons elicit activation within the right IPS and those requiring retrieval of exact arithmetical facts activate the left IPS (e.g.^56–59^). In our experiment, both the primes and the targets were presented in a symbolic notation as tactile Braille digits and Arabic digits. Participants indicated the target quantity while undergoing fMRI scans, thus they activated their symbolic number representation, which, according to the aforementioned research, may be stronger in left IPS.

Taken together, our results show that consciously perceived numerical primes in the tactile or visual modality lead to repetition suppression in left parietal cortex. This provides evidence for the idea that the left parietal cortex hosts neural assemblies that are sensitive to numerical information from different notations (number words or Arabic digits) and modalities (tactile and visual). Whether or not this suggests an abstract number representation remains to be established. Finally, we would like to emphasize that in our study we tested a very peculiar population of subjects, sighted Braille readers. The conclusions we draw should be considered valid only for this particular group.

## Supplementary information

Supplementary file1

## Data Availability

All data generated and analyzed during the study are available from the corresponding author on request.

## References

[1] Dehaene S (1992). Varieties of numerical abilities. Cognition.

[2] Cohen Kadosh R, Cohen Kadosh K, Kaas A, Henik A, Goebel R (2007). Notation-dependent and -independent representations of numbers in the parietal lobes. Neuron.

[3] Dehaene S, Molko N, Cohen L, Wilson AJ (2004). Arithmetic and the brain. Curr. Opin. Neurobiol..

[4] Eger E, Sterzer P, Russ MO, Giraud A-L, Kleinschmidt A (2003). A supramodal number representation in human intraparietal cortex. Neuron.

[5] Kouider S, Dehaene S (2009). Subliminal number priming within and across the visual and auditory modalities. Exp. Psychol..

[6] Piazza M, Pinel P, Le Bihan D, Dehaene S (2007). A magnitude code common to numerosities and number symbols in human intraparietal cortex. Neuron.

[7] Piazza M, Mechelli A, Price CJ, Butterworth B (2006). Exact and approximate judgements of visual and auditory numerosity: an fMRI study. Brain Res..

[8] Teichmann L, Grootswagers T, Carlson T, Rich AN (2018). Decoding digits and dice with Magnetoencephalography: evidence for a shared representation of magnitude. J. Cogniti. Neurosci..

[9] Cohen Kadosh RC (2005). Are numbers special? the comparison systems of the human brain investigated by fMRI. Neuropsychologia.

[10] Pinel P, Piazza M, Le Bihan D, Dehaene S (2004). Distributed and overlapping cerebral representations of number, size, and luminance during comparative judgments. Neuron.

[11] Naccache L, Dehaene S (2001). The priming method: imaging unconscious repetition priming reveals an abstract representation of number in the parietal lobes. Cereb. Cortex.

[12] Cavdaroglu S, Katz C, Knops A (2015). Dissociating estimation from comparison and response eliminates parietal involvement in sequential numerosity perception. NeuroImage.

[13] Bulthé J, De Smedt B, de Beeck HO (2014). Format-dependent representations of symbolic and non-symbolic numbers in the human cortex as revealed by multi-voxel pattern analyses. NeuroImage.

[14] Eger E (2009). Deciphering cortical number coding from human brain activity patterns. Curr. Biol..

[15] Dehaene S, Spelke E, Pinel P, Stanescu R, Tsivkin S (1999). Sources of mathematical thinking: behavioral and brain-imaging evidence. Science.

[16] Molko N (2003). Functional and structural alterations of the intraparietal sulcus in a developmental dyscalculia of genetic origin. Neuron.

[17] Siuda-Krzywicka K (2016). Massive cortical reorganization in sighted Braille readers. Elife.

[18] Bola Ł (2016). Braille in the sighted: teaching tactile reading to sighted adults. PLoS ONE.

[19] Bola Ł (2017). Structural reorganization of the early visual cortex following Braille training in sighted adults. Sci. Rep..

[20] Bola Ł (2017). Universal visual features might be necessary for fluent reading. A longitudinal study of visual reading in braille and cyrillic alphabets. Front. Psychol..

[21] Rączy K, Czarnecka M, Zaremba D, Izdebska K, Paplińska M, Hesselmann G, Szwed M (2020). A shared code for Braille and Arabic digits revealed by cross-modal priming in sighted Braille readers. Acta Psychol..

[22] Millar S (1997). Reading by Touch.

[23] Van Boven RW, Hamilton RH, Kauffman T, Keenan JP, Pascual-Leone A (2000). Tactile spatial resolution in blind Braille readers. Neurology.

[24] Roggeman C, Verguts T, Fias W (2007). Priming reveals differential coding of symbolic and non-symbolic quantities. Cognition.

[25] Reynvoet B, Brysbaert M (1999). Single-digit and two-digit Arabic numerals address the same semantic number line. Cognition.

[26] Reynvoet B, Brysbaert M, Fias W (2002). Semantic priming in number naming. Q. J. Exp. Psychol. A.

[27] Rączy K, Urbańczyk A, Korczyk M, Szewczyk JM, Sumera E, Szwed M (2019). Orthographic priming in braille reading as evidence for task-specific reorganization in the ventral visual cortex of the congenitally blind. J. Cognit. Neurosci..

[28] Glezer LS, Jiang X, Riesenhuber M (2009). Evidence for highly selective neuronal tuning to whole words in the “visual word form area”. Neuron.

[29] Glezer LS, Kim J, Rule J, Jiang X, Riesenhuber M (2015). Adding words to the brain’s visual dictionary: novel word learning selectively sharpens orthographic representations in the VWFA. J. Neurosci..

[30] Barron HC, Garvert MM, Behrens TE (2016). Repetition suppression: a means to index neural representations using BOLD?. Philos. Trans R. Soc. B.

[31] Dehaene S (2001). Cerebral mechanisms of word masking and unconscious repetition priming. Nat. Neurosci..

[32] Devlin JT, Jamison HL, Matthews PM, Gonnerman LM (2004). Morphology and the internal structure of words. Proc. Natl. Acad. Sci. USA.

[33] Hunziker, H. *Im Auge des Lesers: foveale und periphere Wahrnehmung—vom Buchstabieren zur Lesefreude* [The eye of the reader: foveal and peripheral perception—from letter recognition to the joy of reading] (Transmedia, 2006).

[34] Giraudo H, Grainger J (2001). Priming complex words: Evidence for supralexical representation of morphology. Psychon. Bull. Rev..

[35] Nakamura K, Dehaene S, Jobert A, Le Bihan D, Kouider S (2007). Task-specific change of unconscious neural priming in the cerebral language network. Proc. Natl. Acad. Sci. USA.

[36] Notebaert K, Pesenti M, Reynvoet B (2009). The neural origin of the priming distance effect: distance-dependent recovery of parietal activation using symbolic magnitudes. Hum. Brain Mapp..

[37] Kriegeskorte N, Simmons WK, Bellgowan PS, Baker CI (2009). Circular analysis in systems neuroscience: the dangers of double dipping. Nat. Neurosci..

[38] Hesselmann G, Darcy N, Sterzer P, Knops A (2015). Exploring the boundary conditions of unconscious numerical priming effects with continuous flash suppression. Conscious. Cogn..

[39] Hesselmann G, Knops A (2014). No conclusive evidence for numerical priming under interocular suppression. Psychol. Sci..

[40] Lorch RF, Myers JL (1990). Regression analyses of repeated measures data in cognitive research. J. Cognit. Res..

[41] Cohen Kadosh R (2011). Specialization in the human brain: the case of numbers. Front. Hum. Neurosci..

[42] Lyons IM, Ansari D, Beilock SL (2012). Symbolic estrangement: evidence against a strong association between numerical symbols and the quantities they represent. J. Exp. Psychol. Gen..

[43] Shuman M, Kanwisher N (2004). Numerical magnitude in the human parietal lobe: tests of representational generality and domain specificity. Neuron.

[44] Lyons IM, Ansari D, Beilock SL (2015). Qualitatively different coding of symbolic and nonsymbolic numbers in the human brain. Hum. Brain Mapp..

[45] Fias W, Lammertyn J, Reynvoet B, Dupont P, Orban GA (2003). Parietal representation of symbolic and nonsymbolic magnitude. J. Cognit. Neurosci..

[46] Stephan DN, Koch I (2010). Central crosstalk in task switching: Evidence from manipulating input-output modality compatibility. J. Exp. Psychol. Learn. Mem. Cogn..

[47] Stephan DN, Koch I (2011). The role of input-output modality compatibility in task switching. Psychol. Res..

[48] Schaeffner S, Koch I, Philipp AM (2018). Sensory-motor modality compatibility in multitasking: the influence of processing codes. Acta Physiol. (Oxf).

[49] Verguts T, Fias W (2004). Representation of number in animals and humans: a neural model. J. Cognit. Neurosci..

[50] Rivera SM, Reiss AL, Eckert MA, Menon V (2005). Developmental changes in mental arithmetic: evidence for increased functional specialization in the left inferior parietal cortex. Cereb. Cortex.

[51] Cantlon JF, Brannon EM, Carter EJ, Pelphrey KA (2006). Functional imaging of numerical processing in adults and 4-y-old children. PLoS Biol..

[52] Piazza M, Izard V, Pinel P, Le Bihan D, Dehaene S (2004). Tuning curves for approximate numerosity in the human intraparietal sulcus. Neuron.

[53] Cohen L, Dehaene S (1996). Cerebral networks for number processing: evidence from a case of posterior callosal lesion. Neurocase.

[54] Dehaene S, Cohen L (1991). Two mental calculation systems: a case study of severe acalculia with preserved approximation. Neuropsychologia.

[55] Andres M, Seron X, Olivier E (2005). Hemispheric lateralization of number comparison. Cognit. Brain Res..

[56] Chochon F, Cohen L, Moortele PVD, Dehaene S (1999). Differential contributions of the left and right inferior parietal lobules to number processing. J. Cognit. Neurosci..

[57] Dehaene S (1996). The organization of brain activations in number comparison: event-related potentials and the additive-factors method. J. Cognit. Neurosci..

[58] Pinel P, Dehaene S, Riviere D, LeBihan D (2001). Modulation of parietal activation by semantic distance in a number comparison task. Neuroimage.

[59] Rickard T (2000). The calculating brain: an fMRI study. Neuropsychologia.

