# Supplementary material for: Tactile to visual number priming in the left intraparietal cortex of sighted Braille readers
Source: Sci Rep. 2020 Oct 16;10:17571. doi: 10.1038/s41598-020-72431-7 (PMC7567860; doi:10.1038/s41598-020-72431-7)
Supplement: Supplementary file 1 — Supplementary file1 [file 41598_2020_72431_MOESM1_ESM.docx]

**Tactile to visual number priming in the left Intraparietal Cortex of sighted Braille readers**

Katarzyna Rączy^1^, Maria Czarnecka^1^, Małgorzata Paplińska^2^, Guido Hesselmann^3^, André Knops^4^, Marcin Szwed^1^

1 Department of Psychology, Jagiellonian University, Krakow, Poland;

2 The Maria Grzegorzewska University, Warsaw, Poland;

3 Department of General and Biological Psychology, Psychologische Hochschule Berlin, Berlin, Germany;

4 Université de Paris, LaPsyDÉ, UMR CNRS 8240

Corresponding Author: Katarzyna Rączy, Department of Psychology, Jagiellonian University, Kraków, Poland, Tel: +48 692 579 487, [raczy.katarzyna@gmail.com](mailto:raczy.katarzyna@gmail.com)

**Supplementary Materials**

**Supplementary Experiment**

To identify IPS ROI in the main experiment we used the data from another experiment performed by the same group of subjects. The details of this experiment are given below.

**Materials and Methods**

*Stimuli*

Numerosities were presented visually as Arabic digits and set of dots and tactually as Braille numbers. Numerosities 2, 4, 6 and 8 were used in the experiment. Visual numbers were presented as images of size 400 × 400 pixels, either as Arabic digits (visual abstract) or sets of dots (visual non-abstract) and were identical to those used by Bulthé (et al., 2014). The stimuli were positioned approximately 100 cm from participants' eyes, and were visible via a mirror attached to the head-coil. Both formats consisted of centered white circles on a black background. Visual symbolic numerosities presented as Arabic digits varied in position and size across trials. Visual non-symbolic numerosities presented as set of dots were controlled for possible visual feature classification (total luminance and total area spanned by the dots, individual item size and inter-item spacing) and were randomly varied across the trials. Braille numbers were presented on a Braille display (a custom made fMRI-compatible Braille display). To avoid sensory adaptation, they were presented randomly on the first, second or third slot of the Braille display.

*Experimental Design*

The experimental design was identical to the one used by Bulthé (et al., 2014). Additionally, to visual symbolic and non-symbolic numerosities, we added Braille numbers (tactile symbolic). In result participants were presented with numerosities 2, 4, 6 and 8 in three formats: visual symbolic, visual non-symbolic and tactile symbolic. Altogether 12 conditions were presented in a block design. There were 8 runs total and each lasted 6 min 40 s. In each run there were 6 blocks total (72 blocks in the whole experiment). Each block started with 8 second fixation cross presentation and ended with a fixation cross lasting randomly 4-6s. Within such a block, the participants were presented with 12 blocks corresponding to 12 experimental conditions (visual symbolic: 2, 4, 6, 8; visual non-symbolic: 2, 4, 6, 8 and Braille: 2, 4, 6, 8), that included 4-6 stimuli of the same numerosity and format but different visual features or different spacing in case of Braille numbers.

A fixed comparison task was used: the participants were asked to compare each of the presented numerosity (2, 4, 6 and 8) with the fixed reference: number 5 and to press a button corresponding to larger or smaller than 5. This task allowed to reach to the underlying magnitude information but also to control for context-dependent effects on the number representations in the comparison.

*Data acquisition*

fMRI data was acquired at the Laboratory of Brain Imaging (LOBI) in Warsaw on a 3 Tesla Siemens MAGNETOM Trio scanner. Functional MR scans were collected using an EPI sequence (48 slices, 2.1 × 2.1 mm in plane voxel size, slice thickness 2 mm, interslice gap 1 mm, repetition time (TR) = 3000 ms, echo time (TE) = 30 ms, flip angle = 90, 104 × 104 matrix). 3D T1-weighted MPRAGE images (182 slices, resolution 0.98 × 0.98 × 1.2 mm, TR = 9.6 ms, TE = 4.6 ms, 256 × 256 acquisition matrix) were also acquired for each subject. A 32-channel head coil was used.

*Data analysis*

All fMRI data were analyzed using the SPM12 software package. (http://www.fil.ion.ucl.ac.uk/spm/software/spm12/). Using Fourier phase shift interpolation, all the acquired functional volumes were corrected to the first slice for EPI distortion and slice acquisition time; they were subsequently realigned using rigid body transformations to correct for head movements and normalized to the standard adult template (MNI space). Functional data were finally smoothed with a 6 mm (FWHM) Gaussian kernel. The hemodynamic activity for each condition 1) tactile: 2, 4, 6, 8; 2) visual symbolic: 2, 4, 6, 8; 3) visual non-symbolic: 2, 4, 6, 8 and six estimated movement parameters as regressors were first modeled within a general linear model for each subject. In the second level analysis, we carried out a random-effects ANOVA analysis for the group, we applied an uncorrected voxel wise threshold of p < .001.

For the localizer in the main experiment, we compared activation of all numerosities across all formats versus rest.

**
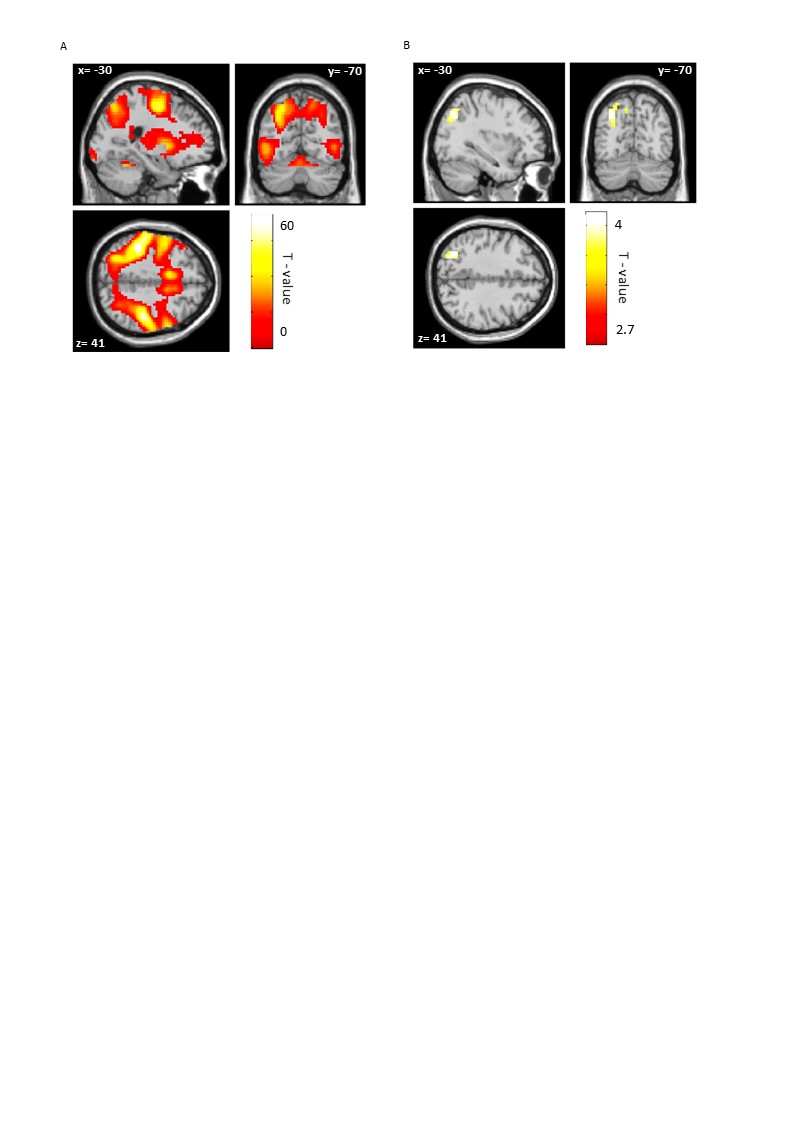
**

**Supplementary Figure 1. Whole-brain analysis (A)** using an inclusive tactile-to-visual and visual-to-visual number mask (All number trials > Rest contrast). **(B) Repetition suppression** for numerical repetition priming with tactile primes (Braille digits) to visual targets (Arabic digits) in Different vs. Same condition masked with All number trials vs. Rest contrast. Threshold p < .001 voxel-wise (uncorrected), p < .01 cluster-wise (FWE-corrected).
